# Clinical significance of large rearrangements in *BRCA1* and *BRCA2*

**DOI:** 10.1002/cncr.27556

**Published:** 2012-04-27

**Authors:** Thaddeus Judkins, Eric Rosenthal, Christopher Arnell, Lynn Anne Burbidge, Wade Geary, Toby Barrus, Jeremy Schoenberger, Jeffrey Trost, Richard J Wenstrup, Benjamin B Roa

**Affiliations:** Myriad Genetic LaboratoriesSalt Lake City, Utah

**Keywords:** *BRCA1* gene, *BRCA2* gene, gene rearrangement, mutation frequency, hereditary breast and ovarian cancer syndrome

## Abstract

**BACKGROUND:** Current estimates of the contribution of large rearrangement (LR) mutations in the *BRCA1* (breast cancer 1, early onset) and *BRCA2* (breast cancer 2, early onset) genes responsible for hereditary breast and ovarian cancer are based on limited studies of relatively homogeneous patient populations. The prevalence of *BRCA1/2* LRs was investigated in 48,456 patients with diverse clinical histories and ancestries, referred for clinical molecular testing for suspicion of hereditary breast and ovarian cancer. **METHODS:** Sanger sequencing analysis was performed for *BRCA1/2* and LR testing for deletions and duplications using a quantitative multiplex polymerase chain reaction assay. Prevalence data were analyzed for patients from different risk and ethnic groups between July 2007 and April 2011. Patients were designated as “high-risk” if their clinical history predicted a high prior probability, wherein LR testing was performed automatically in conjunction with sequencing. “Elective” patients did not meet the high-risk criteria, but underwent LR testing as ordered by the referring health care provider. **RESULTS:** Overall *BRCA1/2* mutation prevalence among high-risk patients was 23.8% versus 8.2% for the elective group. The mutation profile for high-risk patients was 90.1% sequencing mutations versus 9.9% LRs, and for elective patients, 94.1% sequencing versus 5.9% LRs. This difference may reflect the bias in high-risk patients to carry mutations in *BRCA1*, which has a higher penetrance and frequency of LRs compared with *BRCA2*. There were significant differences in the prevalence and types of LRs in patients of different ancestries. LR mutations were significantly more common in Latin American/Caribbean patients. **CONCLUSIONS:** Comprehensive LR testing in conjunction with full gene sequencing is an appropriate strategy for clinical *BRCA1/2* analysis.

Germline mutations in the *BRCA1* (breast cancer 1, early onset) and *BRCA2* (breast cancer 2, early onset) genes are the most important cause of hereditary breast and ovarian cancer (HBOC).^1^ Women with inherited mutations in either of these genes have a lifetime risk of up to 87% for breast cancer and up to 44% for ovarian cancer.^2^, ^3^ Male mutation carriers have significantly increased risks for breast and prostate cancer, and carriers of both sexes have an increased risk for pancreatic cancer.^4^ It is estimated that approximately 1 in 300 individuals carry mutations in *BRCA1* or *BRCA2*, translating to 1,000,000 carrier men and women in the United States alone.^5^, ^6^ The identification of mutations in at-risk patients has significant clinical utility in guiding medical management strategies that can lead to better patient outcomes through cancer prevention, early detection, and treatment of malignancies.^7^ As a result, guidelines from the National Comprehensive Cancer Network and other professional societies recommend clinical analysis of *BRCA1*/*2* for patients who meet specified personal and family cancer history criteria.^8^

The majority of *BRCA1/2* mutations consist of single base changes or deletions/insertions of small numbers of bases that result in protein truncation, disruption of messenger RNA processing, or amino acid substitutions that have significant impact on protein function. These mutations are readily detectable by standard methods for Sanger DNA sequencing of polymerase chain reaction (PCR)-amplified gene segments. A minority of mutations in the *BRCA1*/*2* genes are large rearrangements (LRs) of DNA segments that disrupt gene function. These primarily consist of deletions and duplications of 1 or more exons. These LRs are mostly refractory to detection by PCR-based sequencing, requiring alternative assays such as Southern blotting, multiplex ligation-dependent probe amplification, quantitative PCR, or comparative genomic hybridization.^9^ Recently, it has been demonstrated that massively parallel sequencing technologies have the capability to detect LRs along with sequencing mutations, without requiring additional assays.^10^–^12^ However, adapting next-generation sequencing platforms to optimize detection of LRs may still require additional laboratory and informatics resources beyond those required to determine the sequence data. Furthermore, standards and guidelines for LR testing using massively parallel sequencing platforms have yet to be established in the clinical setting.

Because the detection of LRs requires resources beyond those required for standard sequence analysis, it is important to establish the relative prevalence of this type of mutation in *BRCA1/2* to inform decisions about LR testing for individual patients. In a broader sense, this data can also contribute to the development of consensus regarding whether LR testing should be routinely included as a component of clinical testing for all patients at risk for HBOC. To date, the majority of studies reporting on the detection of LRs in *BRCA1*/*2* have focused on relatively small numbers of individuals of specific ancestries. In addition, some studies have targeted LR testing to very high-risk patients already known to be negative for mutations detected with sequencing, without providing prevalence estimates of sequencing versus LR mutations overall.^13^, ^14^ Estimates of the percentage of *BRCA1/2* mutations that are LRs have ranged from 0% to 40%.^13^

Our laboratory first began limited LR testing in August 2002, using a 5-site Large Rearrangement Panel (LRP) of targeted PCR reactions to detect 5 recurrent LRs in *BRCA1* that had been observed primarily in European populations.^15^ We subsequently developed an additional technology for comprehensive detection of LRs in both *BRCA1* and *BRCA2*. The BRAC*Analysis* Rearrangement Test (BART) is a quantitative multiplex endpoint PCR assay that detects all large deletions and duplications across the coding regions and promoters of *BRCA1* and *BRCA2*. This clinical test has been available since August 2006, wherein BART testing has been performed automatically in conjunction with sequencing for patients meeting criteria indicating a high probability of carrying a *BRCA1/2* mutation. BART has also been performed for patients who do not meet these high-risk criteria, but for whom the test has been ordered on an elective basis.

As of December 1, 2011, our laboratory has performed comprehensive LR testing as a clinical service for more than 64,000 patients, allowing us to analyze data sets an order of magnitude larger than those of any previous study. Furthermore, this referral testing cohort is drawn primarily from the multicultural population of the United States, which includes patients of more diverse ancestries than most previously published studies. Here, we characterize the mutation profile of *BRCA1* and *BRCA2* within this large cohort, stratified by mutation type (sequencing vs LR), prior risk, and patient ancestry.

## MATERIALS AND METHODS

### Patients

All patients were referred to Myriad Genetic Laboratories, Incorporated, for clinical analysis of *BRCA1/2* between July 2007 and April 2011. All patient data regarding clinical history and ancestry were obtained by health care provider report on test requisition forms.

Data were analyzed for 2 groups of patients. The “high-risk” group consisted of 25,535 individuals for whom the test ordered was Comprehensive BRAC*Analysis*, and who met clinical criteria predicting a relatively high probability of carrying a mutation in *BRCA1* or *BRCA2*. Simply stated, most patients met these high-risk criteria if they had invasive or in situ breast cancer diagnosed under age 50 years, or ovarian cancer or male breast cancer diagnosed at any age, in conjunction with 2 or more relatives similarly affected (for specific details of the criteria, see https://www.myriadpro.com/BRAC_BART). These patients received BART automatically as part of their testing.

The “elective” group included 22,921 individuals not meeting the high-risk criteria, but for whom BART was ordered as an elective test to be run only if no deleterious mutation was detected by *BRCA1/2* sequencing and the LRP. Patients were only included in the elective group if BART was ordered at the same time as Comprehensive BRAC*Analysis*. Patients were excluded if the BART testing was cancelled for any reason other than a positive result from sequencing or the LRP.

### Test Descriptions

Comprehensive BRAC*Analysis* testing consists of PCR-based, bidirectional Sanger sequencing of *BRCA1* and *BRCA2*. This encompasses ≍5400 base pairs (bp) comprising 22 coding exons and ≍750 bp of flanking introns in *BRCA1*, and ≍10,200 bp comprising 26 coding exons and ≍900 bp of flanking introns in *BRCA2*.^16^ A patient is considered positive for a sequencing mutation if they are confirmed to have a sequence variant that is considered either “deleterious” or “suspected deleterious” based on American College of Medical Genetics guidelines for mutation classification.^17^ Comprehensive BRAC*Analysis* also includes limited testing for 5 LRs in *BRCA1* using breakpoint-specific PCR reactions called the BRAC*Analysis* 5-site LRP.^15^

BART detects deletions and duplications throughout the *BRCA1* and *BRCA2* genes using our laboratory-developed quantitative endpoint multiplex PCR assay. BART uses a set of 12 reactions comprising 11 multiplex PCR reactions containing 9 to 14 amplicons per multiplex, and 1 contamination detection reaction. These amplicons cover coding exons, promoters, and flanking regions for *BRCA1/2*. Data were reviewed by 2 independent reviewers and verified by a PhD laboratory director prior to reporting. A sample was considered to be positive for a LR after a confirmatory second run and completion of quality control measures, such as checking for sequence variants under primer binding sites. Breakpoints are not routinely determined in the course of clinical testing unless the rearrangement occurs in such proximity to an exon that breakpoint analysis is required for interpretation.

### Statistical Comparisons

All statistical comparisons were performed with Minitab 15 Statistical Software.^18^ Differences were tested using a 2-tailed proportions test with 95% confidence interval. Statistically significant differences were determined with Fisher's exact test.^18^

## RESULTS

### Identification of LR Mutations

**Figure 1:**
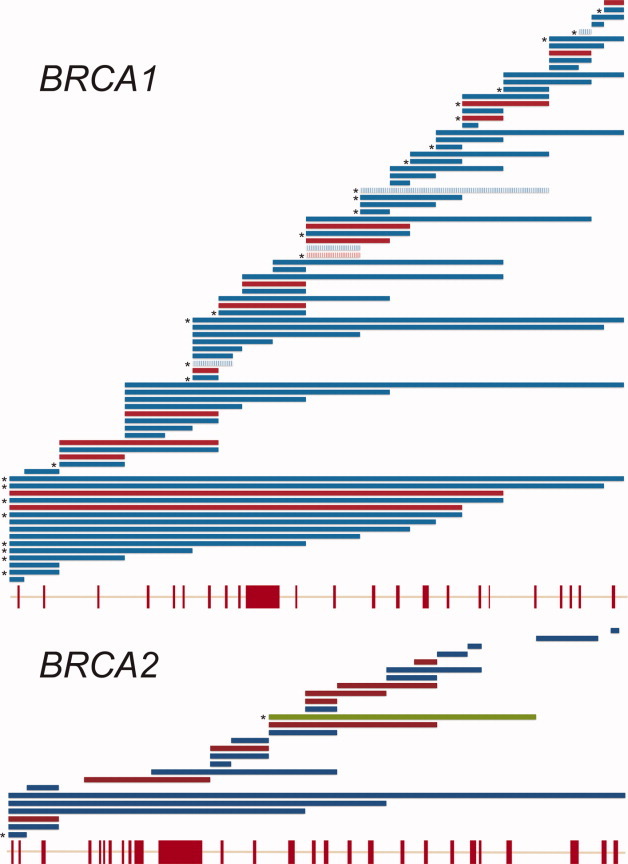
The spectrum of rearrangements detected in *BRCA1* (breast cancer 1, early onset) and *BRCA2* (breast cancer 2, early onset) across all patient samples tested during the given time frame are shown. The extents of large rearrangements relative to a 5′ to 3′ gene structure schematic are depicted. Note that both genes start at exon 1, but that *BRCA1* has no exon 4. Blue bars represent deletions, red bars indicate duplications, and the green bar represents a documented triplication. Five recurrent *BRCA1* rearrangements detected by the Large Rearrangement Panel are indicated with hashed bars. Rearrangements are indicated from the midpoint between affected exons for this schematic; actual breakpoint locations are not implied. Asterisk (*) denotes rearrangements that were observed 5 or more times in this time period.

Among the 48,456 patients included in this study, we detected 81 different LRs in *BRCA1* and 27 LRs in *BRCA2* (Fig .1). These mutations range from deletion or duplication of a single exon to whole-gene deletions of either *BRCA1* or *BRCA2*. We observed a total of 108 LRs, including 84 deletions, 23 duplications, and 1 triplication. The actual number of discrete LR mutations likely exceeds 108 in this group of patients, because many LRs were observed more than once, and the exact LR breakpoints were not routinely defined. Literature reports have documented LRs with different breakpoints that manifest deletion or duplication of the same exons.^19^ For example, we observed at least 2 different versions of *BRCA1* deletion of exons 8-9, one of which is detected with the LRP and the other requiring BART (Fig .1). We have also documented 2 versions of *BRCA1* deletion of exons 1-2, which are distinguishable due to the PCR primer coverage in this region. Aside from a small number of very common LRs, the majority of LR mutations were observed in fewer than 10 patients, with close to half being found in only a single individual (data not shown).

### Prevalence of LR Mutations

In order to establish the prevalence of LRs as a fraction of all mutations in *BRCA1*/*2*, we analyzed results from 2 defined subsets of patients for whom we were able to track all outcomes from the sequencing, LRP, and BART tests. The high-risk group included 25,535 patients who met the clinical criteria described in the methods section, and for whom BART testing was run concurrently with sequencing and LRP assays. The elective group consisted of 22,921 patients for whom BART was ordered electively at the same time as sequencing and the LRP, wherein BART was run only if the sequencing and LRP testing was negative. The overall *BRCA1*/*2* mutation positive rate was 23.8% for the high-risk versus 8.2% for the elective groups (Table 1). LRs detected with either the LRP assay or BART account for 9.9% of mutations in the high-risk group, compared to 5.9% in the elective group (Table 1). The higher proportion of LRs among mutations found in the high-risk group may be partially explained by the finding that 90% of all LRs were observed in *BRCA1*. This was coupled with a substantial enrichment for *BRCA1* mutations of all types in the high-risk group, 66% versus 34%, compared to 48% versus 52% in the elective group, for *BRCA1* versus *BRCA2*, respectively (*P* < .001).

**Table 1:** Mutation Prevalence by Risk Group^a^

				Sequence Mutations		LR Mutations	
	Tests	Pos Rate^b^	B1:B2 Ratio^b^	Seq Pos Rate^b^	B1:B2 Ratio^b^	LR Pos Rate^b^	B1:B2 Ratio	LR % of Positives^b^	BART:LRP Ratio
Elective Group	22,921	8.2%	48%:52%	7.8%	45%:55%	0.5%	90%:10%	5.9%	73%:27%
High-Risk Group	25,535	23.8%	66%:34%	21.5%	63%:37%	2.4%	91%:9%	9.9%	76%:24%

BART indicates BRAC*Analysis* Rearrangement Test; LR, large rearrangement; LRP, Large Rearrangement Panel; Pos, positive; Seq, sequence.

a *BRCA1* and *BRCA2* mutation prevalence for the high-risk and elective groups are based on tests performed within the timeframe of July 2007 to April 2011. Data specific to sequence mutations and LR mutations appear below the designated headings. This table also includes ratios of *BRCA1*:*BRCA2* within sequencing mutations and LR.

b *P* values (*P* < .001 Fisher's exact test) are based on comparison of the values from the elective and high-risk groups.

### Influence of Ancestry on Prevalence and Profile of LR

In order to investigate the mutation profile by ancestry, we selected subsets of patients from both the high-risk and elective groups for whom a single ancestry was reported on the test requisition form (Table 2). Patients for whom multiple ancestries were reported were excluded from this analysis. We observed the highest overall mutation detection rates in patients of African and Latin American/Caribbean ancestry in both the high-risk and elective BART groups. The lowest positive rates were observed for Asian and Native American ancestries in the elective group, and Ashkenazi and Native American patients in the high-risk group.

**Table 2:** Risk and Ancestry Summary^a^

Elective Group	Tests	Pos Rate	Seq Positive	LR Positive	Seq Pos Rate	LR Pos Rate	Seq % of Positives	LR % of Positives	BART %: LRP %
African	947	11.5%	108	1	11.4%	0.1%	99.1%	0.9%	100%:0%
Ashkenazi	250	11.2%	28	0	11.2%	0.0%	100.0%	0.0%	n/a
Asian	661	6.8%	43	2	6.5%	0.3%	95.6%	4.4%	100%:0%
Central/Eastern Europe	971	8.5%	79	4	8.1%	0.4%	95.2%	4.8%	75%:25%
Latin American/Caribbean	830	11.4%	80	15	9.6%	1.8%	84.2%	15.8%^b^	93%:7%
Native American	74	5.4%	4	0	5.4%	0.0%	100.0%	0.0%	n/a
Near East/Mideast	178	9.6%	17	0	9.6%	0.0%	100.0%	0.0%	n/a
None specified only	2772	9.2%	232	22	8.4%	0.8%	91.3%	8.7%	73%:27%
Western/Northern Europe	13,644	7.5%	961	60	7.0%	0.4%	94.1%	5.9%	67%:33%
High-Risk Group	Tests	Pos Rate	Seq Positive	LR Positive	Seq Pos Rate	LR Pos Rate	Seq % of Positives	LR % of Positives	BART %: LRP %
African	1767	29.4%	476	43	26.9%	2.4%	91.7%	8.3%	98%:2%
Ashkenazi	676	12.7%	82	4	12.1%	0.6%	95.3%	4.7%	75%:25%
Asian	552	23.9%	125	7	22.6%	1.3%	94.7%	5.3%	100%:0%
Central/Eastern Europe	1716	24.5%	386	34	22.5%	2.0%	91.9%	8.1%	71%:29%
Latin American/Caribbean	1560	31.2%	383	104	24.6%	6.7%	78.6%	21.4%^b^	99%:1%
Native American	285	17.9%	49	2	17.2%	0.7%	96.1%	3.9%	100%:0%
Near East/Mideast	195	24.6%	40	8	20.5%	4.1%	83.3%	16.7%^c^	100%:0%
None specified only	3831	23.6%	827	78	21.6%	2.0%	91.4%	8.6%	72%:28%
Western/Northern Europe	12,229	23.1%	2552	271	20.9%	2.2%	90.4%	9.6%	64%:36%

BART indicates BRAC*Analysis* Rearrangement Test; LR, large rearrangement; LRP, Large Rearrangement Panel; n/a, not applicable; Pos, positive; Seq, sequence.

a The mutation profile of *BRCA1* and *BRCA2* sequencing and LR mutations between the high-risk and elective groups are broken down by reported ancestry. Patients reporting multiple ancestries were excluded from this portion of the analysis. Ashkenazi Jewish patients only included those who underwent testing beyond the 3 most common Ashkenazi founder mutations.

b *P* < .001, Fisher's exact test.

c *P* = .15, Fisher's exact test.

There are differences in the prevalence of LRs in *BRCA1/2* for some ancestries (Table 2). This is most striking for patients of Latin American/Caribbean and Near East/Mideast ancestries. The percent of mutations that are LRs in patients reporting Latin American/Caribbean descent is significantly higher compared to most other populations, 21.4% for high-risk patients (*P* < .001) and 15.8% for the elective patients (*P* < .001). A single LR, *BRCA1* deletion exons 9-12, made up 37% of all the LRs found in patients of Latin American/Caribbean ancestry (data not shown). This deletion has been previously described as a founder mutation in patients of primarily Mexican ancestry.^20^

Among other ancestries, we also observed a trend toward an elevated frequency of LRs among high-risk patients of Near East/Mideast descent, constituting 16.7% of all mutations; this high relative prevalence of LRs was not replicated in the elective BART patients. However, this is inconclusive due to the small number of Near East/Mideast patients. Seven different LRs were detected in 8 patients of Near East/Mideast ancestry, indicating that a single founder mutation does not account for the observed high frequency of LRs in the high-risk patients (data not shown). A marked disparity in relative LR prevalence between the elective and high-risk groups was observed in patients with African ancestry (8.3% vs 0.9%). The basis for this large difference is unknown. Ashkenazi Jewish patients undergoing testing beyond screening for the 3 Ashkenazi founder mutations (*BRCA1* 187delAG, *BRCA1* 5385insC, and *BRCA2* 6174delT) had the lowest prevalence of LRs of any group.

Table 2 also illustrates the relative utility of the LRP for patients of different ancestries. Overall, the 5 *BRCA1* LRs detected by the LRP make up approximately 25% of all LRs detected in both the high-risk and elective patients, but there are dramatic differences based on patient ancestry. In patients of Western/Northern European ancestry, the LRP finds 36% of all the LRs. However, the mutations included in the LRP are completely absent or very rare in all non-European ancestries.

Table 3 lists LR mutations observed in 5 or more patients who reported a single ancestry, and which show a statistically significant association with the listed ancestry compared to all other ancestries combined. For example, 76% of the 17 observations of a deletion of the entire *BRCA1* gene have been found in patients reporting Latin American/Caribbean descent, although patients of this ancestry make up only 5.5% of all the patients included in this analysis. A few of these LRs appear to be exclusive to certain ancestries, such as the *BRCA1* duplication exons 18-19, which was found only in patients of African ancestry, and *BRCA1* deletion exons 14-20 and *BRCA2* deletion exons 1-2, seen exclusively in patients of Western/Northern European ancestry. The *BRCA1* deletion exon 22, which is tested in the LRP, was observed only in patients reporting European ancestry. Some of these LRs, such as the 5 *BRCA1* mutations in the LRP, have defined endpoints and were previously characterized as founder mutations. However, the same cannot be said of all LR mutations listed in Table 3. For example, *BRCA1* deletion exons 1-2 shows a strong association with Latin American/Caribbean ancestry, but has also been seen in 26 patients of other ancestries. In the absence of individual breakpoint information, it is not known whether these recurrent observations involve the same or different mutations. There are at least 6 different breakpoints reported for *BRCA1* deletion exons 1-2 in the recent literature.^19^

**Table 3:** Recurrent LRs Associated With Different Ancestries^a^

Mutation	Latin America/Caribbean (6.5%)	All Other Specified Ancestries (93.5%)	*P*^b^
*BRCA1* del entire gene	13 (76%)	4 (24%)	<.001
*BRCA1* del exons 1-2	19 (42%)	26 (58%)	<.001
*BRCA1* del exons 9-12	44 (88%)	6 (12%)	<.001
*BRCA1* del exon 14	5 (71%)	2 (29%)	<.001
*BRCA1* del exons 16-17	16 (76%)	5 (24%)	<.001
	African (7.4%)	All Other Specified Ancestries (92.6%)	*P*^b^
*BRCA1* del exons 1-19	6 (75%)	2 (25%)	<.001
*BRCA1* del exon 8	5 (71%)	2 (29%)	<.001
*BRCA1* dup exons 18-19	15 (100%)	0 (0%)	<.001
	Central/Eastern Europe (7.4%)	All Other Specified Ancestries (92.6%)	*P*^b^
*BRCA1* del exon 22	6 (40%)	9 (60%)	<.001
	Western/Northern Europe (70.8%)	All Other Specified Ancestries (29.2%)	*P*^b^
*BRCA1* dup exon 13	91 (91%)	9 (9%)	<.001
*BRCA1* del exons 14-20	13 (100%)	0 (0%)	.015
*BRCA1* del exon 20	19 (90%)	2 (10%)	.054
*BRCA1* del exons 21-24	15 (94%)	1 (6%)	.052
*BRCA2* del exons 1-2	11 (100%)	0 (0%)	.041

*BRCA1* indicates breast cancer 1, early onset gene; *BRCA2* indicates breast cancer 2, early onset gene; LR, large rearrangement.

a These data include LRs identified at least 5 times among patients who reported only one ancestry. Percentages are given after populations to indicate relative population prevalence for this analysis. Percentages are given after counts to indicate the percentage of total observations of that rearrangement represented by the count.

b Fisher's exact test.

## DISCUSSION

This represents the largest study to date comparing the outcomes of clinical *BRCA1/2* mutation analysis, using both Sanger sequencing and an assay for large genomic rearrangements. In contrast to most previous studies, our cohort is representative of the patient population currently targeted for clinical testing in the United States, comprising patients with a wide range of probabilities of carrying a mutation, and inclusive of diverse ancestries. This data provides a framework for assessing the overall importance of LRs for clinical *BRCA1/2* analysis.

Among 25,535 patients meeting higher-risk criteria, *BRCA1/2* mutations were detected in approximately 23.8% of patients, versus 8.2% in the 22,921 elective group of patients. These findings indicate that the high-risk clinical history criteria were effective in identifying patients with higher mutation probabilities. LRs made up 9.9% of all mutations in high-risk patients, breaking down to 14.0% of all *BRCA1* mutations and 2.6% of all *BRCA2* mutations. In patients who had BART as an elective test, LRs made up 5.9% of all mutations, breaking down to 11.8% of *BRCA1* and 1.2% of *BRCA2* mutations. These LR prevalence rates are within the wide ranges reported in previous studies for patients of various nationalities.^13^

We found a higher prevalence of LRs in *BRCA1* compared to *BRCA2*, confirming previous reports.^13^, ^19^ This finding is consistent with the abundance of Alu repeats in *BRCA1*, wherein Alu sequences create “hotspots” for unequal homologous recombination, which can lead to LRs.^21^ It is possible that our current data underestimates LRs in *BRCA2*. Because *BRCA2* mutations are less penetrant than those in *BRCA1*,^5^*BRCA2* carriers may be under-referred for testing due to less striking clinical histories.

The higher prevalence of LR mutations in *BRCA1* versus *BRCA2* partially explains the significantly increased proportion of LR mutations in the high-risk versus elective patients (9.9% vs 5.9%). Patients with more severe personal and family histories tend to carry mutations in *BRCA1* compared with *BRCA2*, consistent with the increased penetrance of *BRCA1* mutations.^5^ However, we cannot rule out other contributing factors, such as potentially higher penetrance for some LR mutations. Notably, the percentage of all *BRCA2* mutations that involved LRs was double in high-risk patients compared to elective BART patients, 2.6% versus 1.0% (*P* = .004). This raises the possibility that *BRCA2* LR mutations could be more penetrant than *BRCA2* sequencing mutations. A smaller difference was observed for *BRCA1* LRs, 14.0% versus 11.8%, which was not statistically significant.

The importance of patient ancestry is most dramatically illustrated by the high prevalence of LR in patients of Latin American/Caribbean ancestry, which is approximately 2-fold higher than the overall population tested. An observed higher proportion of LRs among patients of Near East/Mideast descent was not statistically significant due to small sample size.

Our data indicates that a number of LRs involving the same exonic regions in *BRCA1* and *BRCA2* are strongly associated with a single ancestry, possibly due to founder effects (Table 3). For example, in patients of African ancestry, 3 mutations make up 60.5% of all the LRs detected, with *BRCA1* duplication exons 18-19 alone constituting 33%. In patients of Latin American/Caribbean ancestry 5 mutations make up 81.5% of all the LRs detected, with a single LR, *BRCA1* deletion exons 9-12, comprising 37%. It seems likely that some of the LRs seen in patients of more than one ancestry are not the same mutation in all cases, and mutations that share the same breakpoints may actually be more uniformly limited to a single ancestry. Furthermore, these ancestry designations are extremely broad, and previous reports of LR in *BRCA1* and *BRCA2* have suggested the presence of individual, highly prevalent LR founder mutations in more narrowly defined groups. For example, the *BRCA1* deletion exons 9-12 mutation has been previously reported as extremely common in a group of Hispanic patients of primarily Mexican origin.^20^ However, subsequent studies did not identify any patients of Colombian descent who carry this mutation,^22^ suggesting that this individual mutation may not contribute significantly to the *BRCA1* mutation spectrum in the Latin American/Caribbean population overall.

Our laboratory first began testing for LRs, using the LRP assay, which is a set of PCR reactions designed to detect 5 LRs in *BRCA1* that had been previously identified in the literature. At that time, almost all *BRCA1* and *BRCA2* testing had been performed in patients of European descent, so it is not surprising that the LRP has a much higher sensitivity for the detection of LRs in patients of European descent than in those of other ancestries. In patients of Western/Northern European ancestry, the LRP detects approximately one-third of all LRs versus one-fourth in our total testing population, which remains biased toward patients of European ancestry. Notably, the LRP detected only 3 of 182 total LRs detected in all patients of non-European ancestries combined. These results indicate that the current LRP is an inadequate substitute for comprehensive LR testing and illustrate the pitfalls associated with designing genetic assays based on data from patients of insufficiently diverse ancestries.

In summary, we have demonstrated that LRs comprise a significant fraction, between 6% and 10%, of all clinically significant mutations in *BRCA1* and *BRCA2*. We have also documented considerable variation in the prevalence and profile of LR based on patient ancestry. These observations demonstrate the challenges associated with selectively triaging LR testing to subsets of patients, especially considering that we still have limited data available for patients of many non-European ancestries. With this in mind, it is appropriate to consider the routine inclusion of assays for the comprehensive detection of LRs as part of routine testing for *BRCA1* and *BRCA2* testing for all patients at risk for HBOC. As clinical genetics laboratories continually seek to optimize technologies for mutation detection, with a current focus on massively parallel sequencing platforms, an assessment of performance characteristics in regards to LR detection should be an important part of this process.

## FUNDING SOURCES

This work was funded by Myriad Genetic Laboratories, Incorporated.

**CONFLICT OF INTEREST DISCLOSURE**

All authors are employees of Myriad Genetic Laboratories, Incorporated, and as their compensation, receive salaries and stock options.
